# Prediction and Risk Evaluation for Surgical Intervention in Small Bowel Obstruction †

**DOI:** 10.3390/jcm15010297

**Published:** 2025-12-30

**Authors:** Timur Buniatov, Matthias Maak, Anne Jacobsen, Franziska Czubayko, Axel Denz, Christian Krautz, Georg F. Weber, Robert Grützmann, Maximilian Brunner, Anke Mittelstädt

**Affiliations:** Department of General and Visceral Surgery, University Hospital Erlangen, Friedrich-Alexander-Universität Erlangen-Nürnberg (FAU), Krankenhausstrasse 12, 91054 Erlangen, Germany

**Keywords:** SBO, ileus, emergency, prediction, surgery

## Abstract

**Background/Objectives**: Small bowel obstruction (SBO) is a common surgical emergency associated with significant morbidity and mortality. This retrospective analysis aimed to identify key predictors for the need for surgery in SBO management and to develop a simple clinical risk score to support decision-making. **Methods:** This retrospective study included 285 patients treated for SBO at the University Hospital Erlangen from 2018 to 2022. Pretherapeutic clinical, laboratory, and imaging data, as well as treatment details and outcome parameters were assessed and analyzed using univariate and multivariate logistic regression to identify significant predictors for the need of surgery. A weighted point-based risk score was then derived from the final model, and its discriminative performance was evaluated using receiver operating characteristic (ROC) analysis. **Results:** Of the 285 patients, 234 (82.1%) underwent surgery and 51 (17.9%) were successfully managed conservatively. Multivariate analysis identified the following independent predictors for surgery: 0–1 previous abdominal operation (OR 4.7, *p* = 0.009), serum albumin ≤ 34 g/L (OR 4.5, *p* = 0.011), free intraperitoneal fluid on imaging (OR 3.6, *p* = 0.015), air–fluid levels on plain abdominal X-ray (OR 3.5, *p* = 0.024) and a transition point on CT (OR 11.4, *p* = 0.002). A weighted score (range 0–6 points) was constructed, assigning 1 point to each of the first four predictors and 2 points to the transition point. The score showed good discrimination for predicting the need for surgery (AUC 0.874). Using a cut-off of ≥3 points, sensitivity was 96.2% and specificity 64.7%. The observed proportion of patients requiring surgery increased from 21.4% in the low-risk group (0–2 points) to 88.6% in the intermediate-risk group (3–4 points) and 97.3% in the high-risk group (5–6 points). **Conclusions:** The proposed predictors and the weighted risk score may support bedside decision-making in SBO by distinguishing patients who require surgery from those eligible for conservative management, but they require prospective multicenter validation before routine clinical implementation.

## 1. Introduction

Mechanical small bowel obstruction (SBO) is a critical surgical condition requiring prompt and effective management to prevent severe complications such as bowel ischemia, necrosis, dehydration and aspiration [1]. Morbidity and mortality are particularly high in cases of perforation, with reported mortality rates reaching up to 30% if complications develop [2].

In Germany, approximately 110,000 patients are hospitalized annually for ileus, with an incidence of 108 per 100,000 individuals [3]. SBO accounts for nearly 15% of all surgical admissions from emergency departments and over 20% of these cases require surgical intervention [4,5]. The economic burden is substantial, with estimated costs ranging from $1.7 to $3 billion per year [6,7].

Despite advances in diagnostic imaging and clinical management, decision-making in SBO remains challenging. Clear indicators for immediate surgery—such as cardiovascular instability, sepsis, or perforation—are present in only a minority of patients. Moreover, these signs typically indicate an already advanced disease stage and may appear too late for a timely surgical intervention. Identifying patients who require surgery before complications arise remains a key challenge in SBO management.

Recent studies suggest a paradigm shift toward conservative management in selected SBO cases, reporting successful outcomes in 50% to 80% of patients [8,9,10]. This approach is also reflected in the Bologna guidelines, which recommend conservative treatment in all SBO cases unless signs of strangulation, ischemia, or peritonitis are present [8]. Even in cases of SBO with a “virgin abdomen,” conservative treatment has demonstrated high success rates, with Ghabisha et al. reporting a 90% success rate [11].

While conservative treatment reduces unnecessary laparotomies, it carries the risk of delayed surgery in patients who ultimately require an operation, potentially leading to severe complications. The increasing reliance on non-operative management underscores the need for reliable predictors to guide surgical decision-making. In high-pressure emergency settings, these predictors are essential to avoid missing surgical pathology while minimizing unnecessary operations.

Our study contributes to the growing body of literature by identifying anamnestic, clinical, laboratory and radiological predictors of the need for surgery in SBO and by translating them into a simple weighted risk score for use in the emergency setting. In addition, we specifically explored the impact of previous abdominal surgery by comparing surgical characteristics and intraoperative findings between operated patients with 0–1 versus >1 prior abdominal operations.

## 2. Methods

### 2.1. Study Design

**Figure 1:**
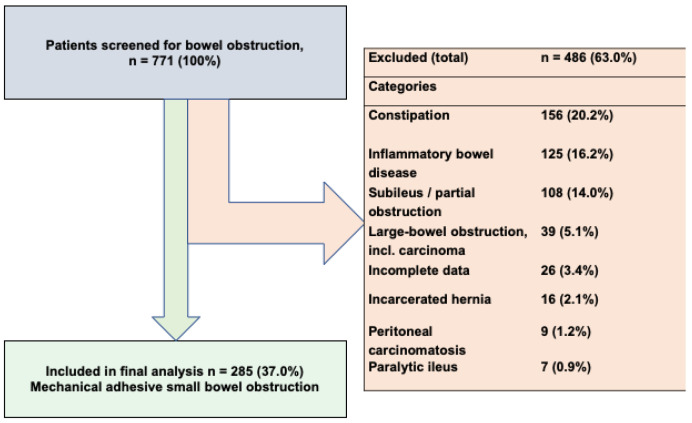
Flow diagram of patient selection for the mechanical small bowel obstruction cohort.

This retrospective study included patients who were hospitalized and treated for small bowel obstruction at the University Hospital Erlangen, Germany, from 2018 to 2022. The initial analysis identified 771 cases of various bowel obstructions, including colonic obstructions, Crohn’s disease, incarcerated hernias, constipation and paralytic ileus. After applying the inclusion and exclusion criteria, 285 patients with confirmed mechanical SBO were selected for final analysis. Inclusion criteria were patients diagnosed and treated for mechanical SBO between 2018 and 2022, aged over 18 years. Exclusion criteria included colonic obstructions, paralytic ileus, malignant bowel obstructions, chronic stenosis from Crohn’s disease or ulcerative colitis, incarcerated hernias, and incomplete medical records. The screening and selection process, including the number and percentage of patients excluded in each category, is summarized in the flow diagram (Figure 1).

Data were extracted from the hospital’s electronic systems, including demographic data, clinical presentation, laboratory values, imaging findings, treatment type (surgical vs. conservative), complications, comorbidities and surgical details.

This study was conducted as a retrospective analysis and was approved by the Ethics Committee of Friedrich-Alexander-Universität Erlangen-Nürnberg (FAU) (Approval Number: 24-414-Br). It was conducted in accordance with national regulations, institutional guidelines, and the principles outlined in the Declaration of Helsinki. Due to the retrospective nature of the study and the use of anonymized patient data, the requirement for informed consent was waived by the Ethics Committee. This manuscript does not contain any patient-identifiable information.

### 2.2. Diagnostic and Therapeutic Algorithm for SBO Management

All patients suspected of small bowel obstruction were initially assessed in our emergency room. Initial diagnostics included a thorough anamnesis, clinical examination, blood tests, abdominal plain X-ray and ultrasound.

If SBO was suspected and the patient was clinically stable, a CT scan was typically performed to determine the cause (e.g., adhesion, malignancies, inflammatory bowel diseases, strangulated internal hernia, etc.), identify the transition point and assess the obstruction level. Immediate surgery was rarely indicated unless the patient was hemodynamically unstable with evidence of an acute, life-threatening abdominal pathology.

Following diagnostic evaluation, treatment decisions (surgical versus conservative management) were made by an experienced senior surgeon. Surgical procedures were performed by experienced surgeons, who determined the extent of surgery based on the underlying cause of the obstruction and intraoperative findings.

Patients considered eligible for conservative management were hemodynamically stable and had no generalized peritonitis, no CT signs of bowel ischemia, closed-loop obstruction, or perforation. These patients were initially managed non-operatively. Conservative treatment at our institution consisted of nasogastric decompression, intravenous fluid and electrolyte replacement, analgesia and close clinical and laboratory monitoring. A water-soluble contrast challenge with oral or nasogastric Gastrografin was used at the discretion of the attending surgeon in patients without clear indications for immediate surgery.

Patients were typically observed for up to 48–72 h under this regimen, in line with internal hospital recommendations, unless earlier surgery was warranted. Indications for conversion to operative treatment included persistent or worsening abdominal pain, development of peritonitis, hemodynamic instability, rising inflammatory markers, CT signs of ischemia or closed-loop obstruction, or failure of contrast medium to progress distally on follow-up imaging.

“Successful conservative treatment” was defined as complete clinical resolution of the SBO episode during the index admission without the need for surgery. In practical terms, this required a soft, non-tender abdomen without peritonitis, normalization or clear improvement of laboratory parameters, cessation of relevant nasogastric output and tolerance of nasogastric tube clamping, resumption of oral intake without vomiting and restoration of bowel function (passage of stool and gas and/or radiological passage of contrast into the colon) together with symptom relief. Long-term outcomes after discharge, including recurrence of obstruction, were not systematically captured and are acknowledged as a limitation of the study.

### 2.3. Statistical Analysis

Statistical analyses were performed using SPSS version 28.0.0.0 (IBM Corp., Armonk, NY, USA) and Jamovi Software version 2.3.28 (Sydney, Australia).

Descriptive statistics and frequency analyses were used to summarize baseline characteristics, clinical presentation, laboratory findings and imaging results. Categorical variables are presented as numbers and percentages, and continuous variables as median and interquartile range (IQR). For the initial comparison between groups, univariate analyses were performed. Student’s *t*-test was used for approximately normally distributed continuous variables, the Mann–Whitney U test for non-normally distributed continuous variables, and the Chi-square test for categorical variables. A *p*-value < 0.05 was considered statistically significant.

Receiver operating characteristic (ROC) curve analysis was used to derive optimal cut-off values for continuous predictors (C-reactive protein, serum albumin, hemoglobin) and to assess the discriminatory performance of the final risk score. In all ROC analyses, the outcome was the need for surgery (operative vs. conservative treatment). Optimal cut-offs were defined by maximizing Youden’s index, yielding thresholds of approximately 22 mg/L for CRP, 34 g/L for albumin and 15.3 g/dL for hemoglobin, which were subsequently used to dichotomize these variables for further analyses. ROC analysis was also applied to determine a clinically meaningful dichotomization of the number of previous abdominal operations (0–1 vs. >1).

To identify independent predictors of surgical intervention, a multivariable binomial logistic regression model was fitted with the need for surgery (operative vs. conservative treatment) as the dependent variable. All variables that were statistically significant in univariate analysis (*p* < 0.05) and considered clinically relevant were entered into the multivariable model using the enter method. A complete-case analysis was performed; only patients with complete data on all candidate predictors were included in the final model, while cases with missing diagnostic data were excluded. In total, 208 of 285 patients with complete information on the relevant predictors were available for the multivariable analysis. For each predictor, odds ratios (ORs) with 95% confidence intervals (CIs) and *p*-values were calculated.

To quantify the overall risk of requiring surgery and to create a clinically applicable tool, we developed a weighted point-based score derived from the final multivariable logistic regression model. For each independent predictor, the corresponding regression coefficient (β = log OR) was used as a measure of its relative contribution to the log odds of surgical treatment. The smallest coefficient (air–fluid levels on abdominal X-ray, β = 1.27) served as the reference. All β-coefficients were divided by this reference value and the resulting ratios were rounded to the nearest integer to obtain an easily applicable point score. Accordingly, low serum albumin (<34 g/L), free intraperitoneal fluid on imaging, air–fluid levels on X-ray and having 0–1 previous abdominal surgery were each assigned 1 point, whereas the presence of a transition point on CT (β = 2.44; highest odds ratio in the model) was assigned 2 points. The total weighted score, therefore, ranged from 0 to 6 points per patient. For each patient, a total score was calculated based on the presence of these predictors, and three risk categories were defined (low, intermediate and high risk), each including both surgically and conservatively treated patients. The discriminative ability of the total weighted score for predicting the need for surgery was assessed using ROC analysis. The optimal cut-off was determined using the Youden index, and the corresponding area under the curve (AUC), sensitivity and specificity at this cut-off were reported.

## 3. Results

### 3.1. Patient Demographics

A total of 285 patients with small bowel obstruction (SBO) were included (median age 68 years, range 20–98; 50.9% female). Of these, 234 patients (82.1%) underwent surgical treatment and 51 (17.9%) were managed successfully with conservative therapy. Baseline characteristics were largely comparable between the operative and conservative groups. There were no significant differences in age, ASA class, BMI or major comorbidities, including arterial hypertension, coronary heart disease, diabetes mellitus and chronic obstructive pulmonary disease (Table 1). As expected, the length of hospital stay was significantly longer in surgically treated patients than in those managed conservatively (median 10 vs. 4 days, *p* < 0.001). Overall, 135 patients (47.4%) had 0–1 previous abdominal operations and 150 (52.6%) had >1 prior operation. The distribution of previous surgery differed markedly between treatment groups (*p* < 0.001). Among patients with 0–1 prior operations, 123/234 patients (52.6%) required surgery, and only 12/51 (23.5%) were treated conservatively. In contrast, in patients with >1 previous abdominal operation, 39/51 patients (76.5%) were managed conservatively and 111/234 patients (47.4%) underwent surgery. The most frequent prior procedures were appendectomy (40.4%), adhesiolysis for ileus (18.6%), urological surgery (18.6%) and colorectal resection (18.2%) (Table 1).

### 3.2. Surgical Characteristics

In total, 234 patients (82.1%) underwent surgery for SBO. Surgical characteristics are summarized in Table 2, stratified by the number of previous abdominal operations (0–1 vs. >1).

Patients with >1 previous surgery were more often operated via primary open laparotomy (*p* = 0.005), whereas laparoscopic adhesiolysis was predominantly used in patients with 0–1 prior operations (*p* < 0.001).

Patients with >1 prior surgery had significantly longer operative times and more complex adhesiolysis, as reflected by higher proportions of “most difficult” adhesiolysis (*p* < 0.001). They also had a higher rate of serosal injuries (*p* < 0.001), consistent with more extensive and dense adhesions (Table 2). Single-band adhesions were more common in patients with 0–1 previous surgery, whereas multiple adhesions predominated in those with >1 previous operation (both *p* < 0.001). In contrast, the rates of small bowel resection, anastomosis and stoma formation did not differ significantly between the two groups.

### 3.3. Conservative Treatment Group

**Figure 2:**
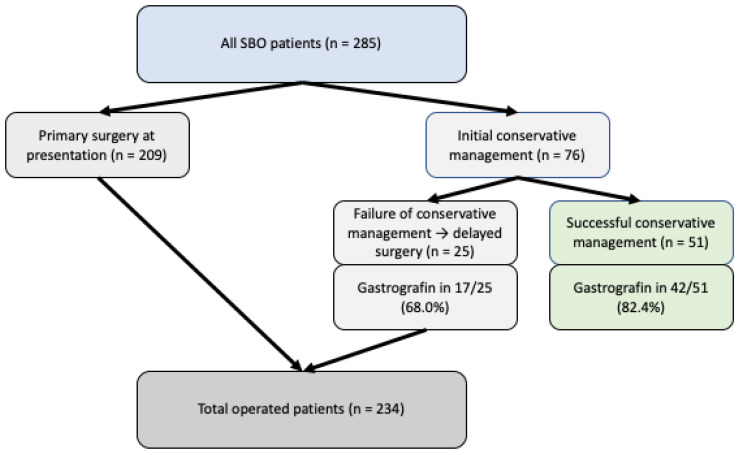
Flow diagram of patient management in small bowel obstruction (SBO).

In our cohort, a total of 76 patients initially underwent conservative management. Conservative therapy was successful in 51/76 patients (67.1%), who recovered without the need for surgery. In contrast, 25/76 patients (32.9%) showed failure of conservative treatment and subsequently required delayed surgical intervention. An oral Gastrografin challenge was performed in 59/76 patients (77.6%) who were treated conservatively. Among these, 42/76 patients (55.3% of all conservatively managed patients) experienced resolution of SBO after Gastrografin administration, whereas 17/76 patients (22.4%) did not respond adequately and ultimately required surgery (Figure 2).

### 3.4. Predictors of Surgical Intervention

In univariate analysis, eight variables were significantly associated with the need for surgery: 0–1 previous abdominal surgery (*p* < 0.001), low serum albumin < 34 g/L (*p* < 0.001), preoperative CRP > 22 mg/L (*p* < 0.001), preoperative hemoglobin > 15.3 g/dL (*p* = 0.026), muscular defense on clinical examination (*p* = 0.003), free intraperitoneal fluid on imaging (*p* < 0.001), air–fluid levels on plain abdominal X-ray (*p* = 0.028) and the presence of a transition point on CT scan (*p* < 0.001).

In the multivariate logistic regression model, five of these remained independent predictors of surgical intervention (Table 3): 0 or 1 previous abdominal surgery (OR 4.7, *p* = 0.009), serum albumin < 34 g/L (OR 4.5, *p* = 0.011), air–fluid levels on X-ray (OR 3.5, *p* = 0.024), free fluid in diagnostic imaging (OR 3.6, *p* = 0.015), and a transition point on CT (OR 11.4, *p* = 0.002).

### 3.5. Risk Assessment

Based on these five independent predictors, we constructed a weighted clinical risk score as described in the Methods. Each of the following variables was assigned 1 point: 0–1 previous abdominal surgery, serum albumin < 34 g/L, air–fluid levels on X-ray and free intraperitoneal fluid on imaging, whereas the presence of a transition point on CT (the strongest predictor) was assigned 2 points. The total score, therefore, ranged from 0 to 6 points.

The observed probability of surgery increased markedly across the predefined risk categories (Table 4). Among patients with 0–2 points (low-risk group), 9/42 (21.4%) required surgery. In those with 3–4 points (intermediate-risk group), 117/132 (88.6%) underwent surgery, while in the high-risk group (5–6 points), 108/111 patients (97.3%) were operated on.

**Figure 3:**
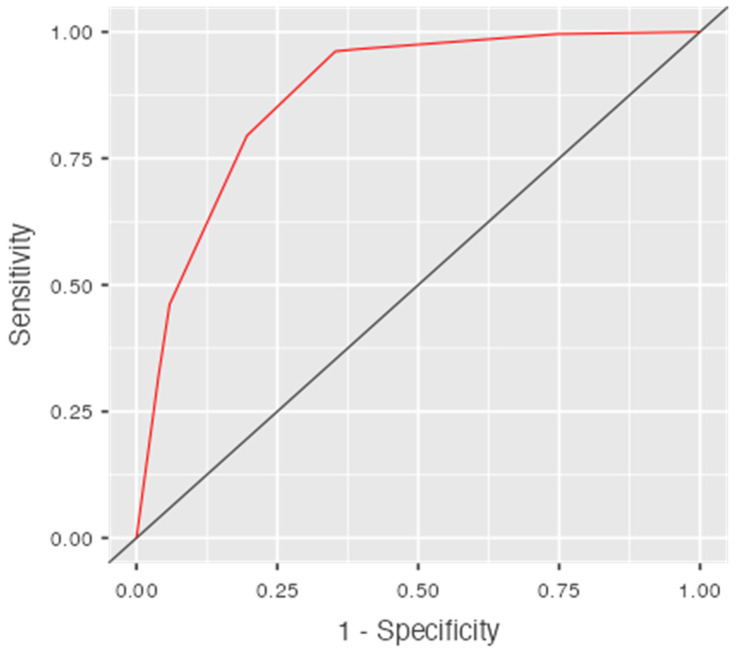
Receiver operating characteristic (ROC) curve of the weighted risk score for predicting the need for surgery in patients with small bowel obstruction. The area under the curve (AUC) is 0.874. The optimal cut-off of ≥3 points (Youden index 0.609) corresponds to a sensitivity of 96.2% and a specificity of 64.7%.

In ROC analysis, the weighted score demonstrated good discriminative ability for predicting the need for surgery, with an AUC of 0.874 (Figure 3). Using the Youden index, an optimal cut-off of ≥3 points was identified, yielding a sensitivity of 96.2% and a specificity of 64.7% (Youden index 0.609).

## 4. Discussion

The timely identification of patients requiring surgical intervention in small bowel obstruction is crucial for optimal management and patient outcomes [12]. Decision-making in these cases remains challenging, even for experienced surgeons, particularly in emergency settings. Delayed treatment is associated with increased morbidity and mortality, as highlighted in studies by Fevang et al. and Schraufnagel et al., emphasizing the importance of prompt surgical management to improve outcomes [13,14].

Our study emphasizes the clinical relevance of specific predictors that facilitate the decision-making process regarding surgical versus conservative treatment. One of the most significant predictors was the number of prior abdominal surgeries. Patients with 0–1 prior surgeries were significantly more likely to require surgical intervention, whereas those with multiple prior surgeries could be treated more often conservatively (OR 4.7, *p* = 0.009).

A plausible biological explanation for the higher surgical rate in patients with 0–1 prior operations is the underlying adhesion pattern. After a single operation, SBO is more likely to be caused by a solitary band adhesion, which is prone to cause strangulation and bowel ischemia. In contrast, patients with multiple previous procedures more often present with diffuse or matted adhesions, which may cause obstruction but less frequently result in volvulus or critical vascular compromise. This concept is supported by previous studies showing that solitary band adhesions are associated with a higher risk of strangulation and bowel necrosis [15]. Meissner et al. reported that single-band adhesions led to strangulation in up to 30% of cases [16]. In addition, Suh and Choi demonstrated that conservative management is often ineffective in patients with single-band adhesions, whereas laparoscopic adhesiolysis can be an effective treatment option in this subgroup [17]. Consistent with these data, laparoscopy in our cohort was more frequently used in patients with few or no previous operations, who are more likely to have solitary adhesions.

At the same time, we fully acknowledge that this association may partly be driven by selection bias. Surgeons may be more reluctant to operate on “hostile abdomens” with multiple previous laparotomies because of the anticipated technical difficulty and higher risk of iatrogenic injury, and therefore may preferentially attempt prolonged conservative treatment in these patients. In other words, the number of prior surgeries may not only reflect a different biological pattern of adhesions, but also influence the treatment strategy chosen by the surgeon. Our observational design does not allow us to disentangle these mechanisms completely.

Nevertheless, our findings are in line with a recent meta-analysis from 2023, including 4638 patients across 31 studies, which identified previous abdominopelvic surgery as a predictor of successful conservative treatment [18]. Similarly, Collom et al. found that previous abdominal surgery predicted conservative therapy success compared with patients without prior operations [19]. Taken together, these data suggest that a history of multiple abdominal procedures may identify a subgroup in whom conservative management is more likely to succeed, although this is likely influenced by both adhesion morphology and surgeon decision-making.

Another key predictor identified in our study was hypoalbuminemia, a widely accessible and cost-effective laboratory marker. Albumin serves as an indicator of a patient’s nutritional status and systemic inflammation. Hypoalbuminemia is well established as a marker of acute–phase response, impaired protein synthesis, and increased vascular permeability—common consequences of severe stress, sepsis, or prolonged bowel obstruction. SBO-induced intraluminal volume shifts, dehydration and inflammatory cytokine release further exacerbate albumin depletion [20,21]. Low albumin levels have been strongly associated with postoperative complications, including impaired wound healing, surgical site infections, prolonged hospital stays and increased mortality [22,23,24,25]. Our findings align with Besler et al., who identified hypoalbuminemia as a significant risk factor for surgery in adhesive SBO (*p* < 0.001), and Sahin et al., who highlighted albumin as a predictor for surgical intervention [26,27]. Given its accessibility and prognostic significance, albumin is an invaluable marker in clinical decision-making for SBO management.

CRP, another widely used inflammatory marker, was identified in univariate analysis as a significant predictor for surgical intervention (*p* < 0.001). However, in multivariate analysis, it approached but did not reach statistical significance (*p* = 0.058). ROC analysis determined a cutoff value of 22 mg/dL as a practical threshold for clinical decision-making. Previous studies have demonstrated CRP’s predictive role in SBO. Cho et al. found elevated CRP levels to be associated with surgical intervention (*p* = 0.028), while Schwentner et al. reported a CRP threshold of ≥75 mg/L as predictive for surgery [28,29]. Issakson et al. also highlighted CRP as an early indicator for surgical need in SBO patients [30]. The predictive value of CRP likely stems from its role in detecting bacterial translocation and systemic inflammation, making it a useful adjunct in SBO risk stratification [31,32].

Instrumental diagnostics remain essential in SBO evaluation, complementing clinical and laboratory findings. Standard diagnostic tools include abdominal radiographs, ultrasound and CT imaging. Our study confirmed that certain radiological findings strongly predict the need for surgery. Free intraabdominal fluid, detected via ultrasound or CT, was one of the strongest predictors for surgical intervention. Zielinski et al. identified free fluid as a significant predictor (*p* = 0.006), a finding corroborated by Tavangari et al. and Ng et al. [33,34,35]. Similarly, Besler et al. demonstrated that free fluid on CT was more prevalent in patients requiring surgery [26]. These results reinforce its value as a reliable indicator of complicated SBO. Air–fluid levels on abdominal X-ray were also strongly associated with surgical intervention in both univariate and multivariate analyses, consistent with prior research by Aldemir et al., who demonstrated a significant correlation between air–fluid levels and the need for surgery [36]. The transition point on CT, observed in 88.1% of our cases, was another critical predictor with the highest OR (OR 11.4, *p* = 0.002). This aligns with studies by Ng et al., O’Leary et al., Tavangari et al. and Scrima et al., all of whom identified the transition point as a key determinant of surgical necessity [34,35,37,38].

A major strength of this study is the relatively large, well-characterized single-center cohort and the comprehensive inclusion of clinical, laboratory and imaging parameters routinely available in the acute setting. By identifying five independent pretherapeutic predictors and integrating them into a weighted point-based risk score, we provide a simple bedside tool that allows stratification of patients with SBO into low-, intermediate- and high-risk categories with good discriminative performance (AUC = 0.874). The clear separation of primary surgery, initial conservative management and surgery after failed conservative therapy further reflects real-world decision pathways and may help to better understand treatment selection in clinical practice.

However, several limitations need to be acknowledged. First, the retrospective design and the fact that the study was conducted at a single tertiary referral center may limit the generalizability of the findings and introduce selection bias, as a considerable proportion of patients presented with advanced disease and were triaged early to surgery. This likely contributed to the substantial imbalance between the surgically and conservatively treated groups (82.1% vs. 17.9%), which may affect model stability and further limit the transferability of our findings to settings with higher rates of non-operative management.

Second, from a statistical perspective, although the prediction model and the derived weighted score showed good discrimination (AUC = 0.874), no formal calibration analysis (e.g., calibration plots or Hosmer–Lemeshow test) and no internal or external validation were performed. In addition, a complete-case analysis was used, meaning that patients with missing diagnostic or laboratory data were excluded from the multivariable model, which may introduce further bias and underlines the exploratory nature of the score.

Another important limitation is the variability and partial incompleteness of imaging data. In particular, ultrasound findings depend strongly on the examiner’s experience, and key CT parameters such as the small bowel feces sign, the grade of obstruction, closed-loop configuration, or standardized signs of ischemia were not systematically recorded or analyzed. Moreover, there was no predefined institutional algorithm for deciding between surgical and conservative management; treatment decisions and the choice of laparoscopic versus open approach were largely based on the judgment, experience and preferences of the attending surgeon. Finally, systematic long-term follow-up, especially for patients treated conservatively, was not available, which precludes a detailed assessment of recurrence rates, late complications and the long-term safety of non-operative management.

Future research should therefore focus on multicenter prospective validation of the proposed risk score in different healthcare settings, including formal calibration and internal/external validation procedures. Further refinement of the model by incorporating additional objective markers such as serum lactate and standardized radiological parameters (e.g., obstruction grade, ischemia signs) appears warranted. In addition, structured long-term follow-up studies of conservatively managed patients are needed to evaluate recurrence, delayed complications and the long-term safety and effectiveness of non-operative treatment strategies in SBO.

## 5. Conclusions

Diagnosing small bowel obstruction and determining the need for surgery remain challenging, even for experienced surgeons. In this single-center cohort, we identified five readily available pretherapeutic predictors independently associated with the need for surgery: having 0–1 previous abdominal operations, hypoalbuminemia, free intraperitoneal fluid on imaging, air–fluid levels on plain X-ray, and a transition point on CT. Based on these variables, we derived a simple weighted risk score (range 0–6 points) that showed good discriminative performance for predicting the need for operative treatment. The proposed predictors and score may support bedside decision-making in SBO by helping to distinguish patients who are likely to require surgery from those who may be suitable for conservative management, thereby potentially reducing unnecessary operations and improving patient outcomes.

## Figures and Tables

**Table 1:** Patients characteristics.

	All Patients (n = 285)	Operative Group (n = 234)	Conservative Group (n = 51)	*p*
**Baseline characteristics**				
**Age (years), median (IQR)**	68 (23)	67 (25.8)	70 (17.5)	0.212
**Age (>68 years), n (%)**	141 (49.5)	110 (47.0)	31 (60.8)	0.075
**Gender, n (%)**				0.362
**Female**	145 (50.9)	122 (52.1)	23 (45.1)	
**Male**	140 (49.1)	112 (47.9)	28 (54.9)	
**ASA, n (%)**				0.289
**I**	30 (10.5)	27 (11.5)	3 (5.9)	
**II**	112 (39.3)	92 (39.3)	20 (39.2)	
**III**	135 (47.4)	107 (45.7)	28 (54.9)	
**IV**	8 (2.8)	8 (3.4)	0 (0.0)	
**ASA I–II vs. III–IV n, (%)**				0.456
**I/II**	142 (49.8)	119 (50.9%)	23 (45.1%)	
**III/IV**	143 (50.2)	115 (49.1)	28 (54.9)	
**BMI (kg/m^2^), median (IQR)**	25.3 (6.1)	25.1 (6.5)	25.3 (5.2)	0.879
**BMI (kg/m^2^)**				0.703
**>25**	144 (50.5)	117 (50.0)	27 (52.9)	
**≤25**	141 (49.5)	117 (50.0)	24 (47.1)	
**Hospital stay (days), median (IQR)**	9 (8)	10 (9)	4 (3)	**<0.001**
**Medical history**				
**Arterial hypertension, n (%)**	143 (50.2)	114 (48.7)	29 (56.9)	0.292
**Coronary heart disease, n (%)**	65 (22.8)	53 (22.6)	12 (23.5)	0.892
**Diabetes, n (%)**	34 (11.9)	28 (12.0)	6 (11.8)	0.968
**Crohn’s disease, n (%)**	17 (6.0)	14 (6.0)	3 (5.9)	0.978
**Ulcerative colitis, n (%)**	7 (2.5)	3 (1.3)	4 (7.8)	**0.006**
**COPD, n (%)**	25 (8.8)	20 (8.5)	5 (9.8)	0.774
**Number of previous surgeries, n (%)**				**<0.001**
**0 or 1 previous surgery**	135 (47.4)	123 (52.6)	12 (23.5)	
**>1 previuous surgery**	150 (52.6)	111 (47.4)	39 (76.5)	
**Type of previous surgery, n (%)**				
**Appendectomy**	115 (40.4)	81 (34.6)	34 (66.7)	**<0.001**
**Colon resections**	52 (18.2)	46 (19.7)	6 (11.8)	0.186
**Gynecological surgery**	31 (10.9)	24 (10.3)	7 (13.7)	0.461
**Ileus operation**	53 (18.6)	34 (14.5)	19 (37.3)	**<0.001**
**Urological surgery**	53 (18.6)	34 (14.5)	19 (37.3)	**<0.001**
**Blood results**				
**Preoperative WBC (10^9^/^L^), median (IQR)**	10.2 (5.8)	10.4 (6.1)	9.0 (4.3)	0.209
**Preoperative albumin ≤ 34 g/L, n (%)**	158 (55.4)	142 (60.7)	16 (31.4)	**<0.001**
**Preoperative CRP > 22 mg/L, n (%)**	87 (30.5)	87 (30.5)	4 (7.8)	**<0.001**
**Preoperative hemoglobin (>15.3 g/dL), n (%)**	61 (21.4)	56 (23.9)	5 (9.8)	**0.026**
**Preoperative creatinine (mg/dL), median (IQR)**	0.9 (0.4)	0.9 (0.48)	0.9 (0.3)	0.949
**Clinical characteristics**				
**Vomiting, n (%)**				
**1×**	65 (22.8)	49 (20.9)	16 (31.4)	0.108
**>1×**	161 (56.5)	138 (59.0)	23 (45.1)	0.070
**Days of constipation, n (IQR)**	1 (2)	0.99 (2.0)	0.84 (1.0)	0.060
**Bloated abdomen, n (%)**	223 (78.2)	186 (79.5)	37 (72.5)	0.277
**Muscular defense, n (%)**	54 (18.9)	52 (22.2)	2 (3.9)	**0.003**
**Imaging techniques**				
Suspicious ultrasound, n (%) *^,^**	175 (90.7)	135 (91.8)	40 (87.0)	0.321
Free fluid in imaging, n (%) *	187 (65.6)	170 (72.6)	17 (33.3)	**<0.001**
Air–fluid level X-ray, n (%) *	178 (76.1)	148 (79.1)	30 (63.8)	**0.028**
Transition point in CT-scan, n (%) *	238 (92.6)	217 (96.0)	21 (67.7)	**<0.001**

* Data incomplete. ASA = American Society of Anesthesiologists Score; BMI = Body Mass Index; WBC = White Blood Cells; CRP = C-Reactive Protein. ** abnormal peristalsis, dilated loops. Percentages refer to column-wise distributions of categorical variables. Continuous variables are reported as median and interquartile range (IQR).

**Table 2:** Surgical Characteristics of the operated patients.

	Operated Patients (n = 234)	0 or 1 Previous Surgery (n = 123)	>1 Previous Surgery (n = 111)	*p*
Surgical approach, n (%)				
open	222 (94.9)	112 (91.1)	110 (99.1)	0.005
started laparoscopically	23 (9.8)	20 (16.3)	3 (2.7)	<0.001
conversion to open procedure (n, % of laparoscopic surgery) *	11 (47.8)	9 (45.0)	2 (66.7)	0.484
Operative time (min), median (IQR)	110 (80)	90 (60)	130 (100)	<0.001
Difficulty of adhesiolysis, n (%)				<0.001
Easy (<60 min)	53 (22.6)	41 (33.3)	12 (10.8)	
More difficult (60–120 min)	90 (38.5)	49 (39.8)	41 (36.9)	
Most difficult (>120 min)	80 (34.2)	23 (18.7)	57 (51.4)	
Cause of ileus, n (%)				
Single adhesion	75 (32.1)	57 (46.3)	18 (16.2)	<0.001
Multiple adhesions	141 (60.3)	50 (40.7)	91 (82.0)	<0.001
Serosal injury, n (%)	86 (36.8)	33 (26.8)	53 (47.7)	<0.001
Enterotomy, n (%)	33 (14.1)	14 (11.4)	19 (17.1)	0.208
Small bowel segmental resection, n (%)	47 (20.1)	23 (18.7)	24 (21.6)	0.577
Anastomosis, n (%)	47 (20.1)	23 (18.7)	24 (21.6)	0.577
Stoma, n (%)	14 (6.0)	7 (5.7)	7 (6.3)	0.843

* Percentages are calculated within columns (i.e., relative to all operated patients in each previous-surgery group), except for conversion to open surgery, where percentages refer to the number of laparoscopic procedures in the respective group.

**Table 3:** Uni and multivariate analysis.

	Operative Group vs. Conservative Treatment Group			
	Univariate	Multivariate		
		OR	95% CI	*p*
**Age (>68 vs. ≤68 years)**	0.075	-	-	-
**Gender (male vs. female)**	0.362	-	-	-
**ASA (I/II vs. III/IV)**	0.456	-	-	
**BMI (kg/m^2^) (>25 vs. ≤25)**	0.703	-	-	-
**Arterial hypertension (no vs. yes)**	0.292	-	-	-
**Coronary heart disease (no vs. yes)**	0.892	-	-	-
**Diabetes (no vs. yes)**	0.966	-	-	-
**0 or 1 previous surgery**	**<0.001**	**4.7**	**1.4–15.1**	**0.009**
**Preoperative albumin ≤ 34 g/L**	**<0.001**	**4.5**	**1.4–14.3**	**0.011**
**Preoperative CRP > 22 mg/L**	**<0.001**	**8.2**	**0.9–71.6**	0.058
**Preoperative hemoglobin (>15.3 g/dL)**	**0.026**	**5.0**	**0.8–29.0**	0.071
**Muscular defense**	**0.003**	**4.6**	**0.4–46.5**	0.197
**Suspicious ultrasound**	0.323	-	-	-
**Free fluid in diagnostic imaging**	**<0.001**	**3.6**	**1.3–10.3**	**0.015**
**Air–fluid levels in X-ray**	**0.028**	**3.5**	**1.2–10.6**	**0.024**
**Transition point in CT scan**	**<0.001**	**11.4**	**2.4–54.5**	**0.002**

ASA = American Society of Anesthesiologists Score; BMI = Body Mass Index; WBC = White Blood Cells; CRP = C-Reactive Protein. Multivariate: Total number: 208 (due to missing data in X-ray or CT diagnostic).

**Table 4:** Observed probability of surgery according to the weighted risk categories.

Risk Group	Score Range	Number of Patients, n	Surgery, n	Risk of Surgery, %
Low risk	0–2 points	42	9	21.4
Intermediate risk	3–4 points	132	117	88.6
High risk	5–6 points	111	108	97.3

## Data Availability

The datasets generated and analyzed during the current study are not publicly available due to the presence of personal information related to patients. However, they are available from the corresponding author upon reasonable request.

## Abbreviations

SBO	Small Bowel Obstruction
CRP	C-Reactive Protein
ROC	Receiver Operating Characteristic
AUC	Area Under the Curve
CT	Computed Tomography
ASA	American Society of Anesthesiologists
